# A new hand-held microfluidic cytometer for evaluating irradiation damage by analysis of the damaged cells distribution

**DOI:** 10.1038/srep23165

**Published:** 2016-03-17

**Authors:** Junsheng Wang, Zhiqiang Fan, Yile Zhao, Younan Song, Hui Chu, Wendong Song, Yongxin Song, Xinxiang Pan, Yeqing Sun, Dongqing Li

**Affiliations:** 1 College of Information and Science Technology, Dalian Maritime University, Dalian, 116026, China; 2 College of Marine Engineering, Dalian Maritime University, Dalian, 116026, China; 3 College of Environmental Science and Engineering, Dalian Maritime University, Dalian, 116026, China; 4 Department of Mechanical & Mechatronics Engineering, University of Waterloo, Waterloo, ON N2L3G1, Canada

## Abstract

Space radiation brings uneven damages to cells. The detection of the distribution of cell damage plays a very important role in radiation medicine and the related research. In this paper, a new hand-held microfluidic flow cytometer was developed to evaluate the degree of radiation damage of cells. The device we propose overcomes the shortcomings (e.g., large volume and high cost) of commercial flow cytometers and can evaluate the radiation damage of cells accurately and quickly with potential for onsite applications. The distribution of radiation-damaged cells is analyzed by a simultaneous detection of immunofluorescence intensity of γ-H2AX and resistance pulse sensor (RPS) signal. The γ-H2AX fluorescence intensity provides information of the degree of radiation damage in cells. The ratio of the number of cells with γ-H2AX fluorescence signals to the total numbers of cells detected by RPS indicates the percentage of the cells that are damaged by radiation. The comparison experiment between the developed hand-held microfluidic flow cytometer and a commercial confocal microscope indicates a consistent and comparable detection performance.

The effect of radiation on human health is a key issue, especially with the increase in human activities in the exploration of outer space^1^,^2^,^3^,^4^,^5^,^6^. Efforts have been made to study some biological materials in human body whose changes can be used to evaluate the degree of radiation damage. Currently, γ-H2AX is considered as one of the promising biomarkers for cell radiation damage^7^,^8^,^9^,^10^,^11^,^12^,^13^. γ-H2AX is formed from histone H2AX after being radiated at discrete nuclear foci that contain DNA repair factors like 53BP1. The quantities of these foci in cells are regarded as a valuable parameter to indicate the radiation damage. The amount of γ-H2AX can be detected by immunofluorescence methods using primary γ-H2AX antibody and FITC fluorescent secondary antibody^14^,^15^,^16^,^17^, where the fluorescent intensity of FITC dye is proportional to the quantities of γ-H2AX foci and hence the radiation damage in cells.

However, space radiation brings uneven damages to cells, and the irregular distribution of radiation damage plays a very important role in radiation medicine and research^18^,^19^,^20^. Therefore, the immunofluorescence intensity of γ-H2AX alone is unable to provide the complete information to evaluate the radiation damage. At least two parameters are needed for this purpose: one is the immunofluorescence intensity to measure the degree of damage for the damaged cells, and the other is the ratio of the number of damaged cells to the total number of cells in the sample under a given radiation dose.

The most common method of detecting fluorescence intensity of immunofluorescence is the use of flow cytometer^21^. The distribution of the foci with γ-H2AX can only be measured by a confocal microscope. However, these commercial equipments require well-trained operators, involve complex operation procedures, and consume large volume samples and reagents. Furthermore, their large volume prevents them from applications for on-site or point-of-care detection^22^,^23^.

The microfluidic chip technology enables the development of portable flow cytometers with many advantages such as handling small volume of sample and quick detection^24^,^25^,^26^,^27^,^28^,^29^,^30^,^31^. However, in order to achieve high sensitivity in fluorescent detection, microfluidic flow cytometers usually contain embedded optical fibers, which significantly complicates the design of the device and increases its cost. Recently, a flow cytometer with a disposable microfluidic chip has been developed to detect the fluorescence intensity of immunofluorescence of γ-H2AX^32^. However, this device can only count the cells stained with fluorescence dyes and cannot obtain the number ratio of the damaged cells to all the cells.

Therefore, in order to completely evaluate radiation damage, a new hand-held microfluidic flow cytometer was developed in this paper. This device can measure two parameters: one is the number ratio of the damaged cells to all the cells under a given radiation dose, the other is the immunofluorescence intensity of damage cells. A resistive pulse sensor (RPS) is used to measure the total number of cells. A miniaturized fluorescent detection module is used to detect the immunofluorescence intensity of γ-H2AX in cells and hence count the number of radiation damaged cells. The degree of radiation damage can be reflected by the distribution of the immunofluorescence intensity of γ-H2AX in the damaged cells. The number ratio of the cells with fluorescent dye to all the cells in the sample presents the percentage of the cells that are damaged. In order to evaluate the performance of the developed hand-held microfluidic flow cytometer, lymphocyte cells are taken as samples. The results from the developed hand-held microfluidic flow cytometer are compared with those from a commercial confocal microscope.

## Methods and Materials

### Signal detection system

**Figure 1:**
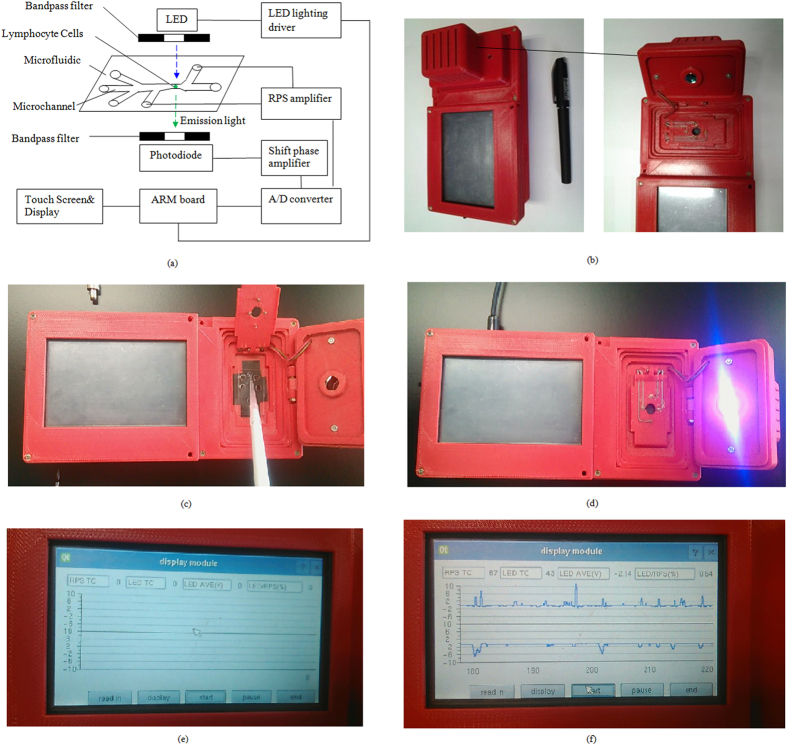
(**a**) Schematic diagram of the microfluidic flow cytometer with fluorescence and RPS detection. (**b**–**f** )Pictures of the hand-held microfluidic flow cytometer.

Resistance pulse sensor (RPS) is based on Coulter principle and can be used to count the number of cells by detecting the impedance change when a cell is passing through a small sensing gate. The signal intensity of RPS is proportional to particle size for a given sensing gate. The fluorescent signals are detected by using a self-designed miniature optical detection system as shown in Fig. 1. The detection system is comprised of a microfluidic chip platform, an excitation light source, a photo-detector, optical filters, and a data acquisition and processing unit. In fluorescence detection part, according to the excitation and emission spectrum of lymphocyte cells stained by FITC fluorescent dyes (the excitation peak of 492 nm and emission peak of 525 nm), a LED (LZ1-10B200, cental wavelength of 485 nm, LED Engin, Inc., San Jose, CA, USA) is chosen as the excitation light source. In order to obtain a stable light output from LED, a LED lighting driver (STCS2ASPR, STMICROELECTRONICS, GENEVA, Switzerland) is used to drive the LED. A photodiode (S8745-01, Hamamatsu, Bridgewater, NJ) is used to detect the fluorescence. The output voltage of the photodiode is positively correlated to the fluorescence intensity. In order to block other scattered light, an emission filter (ET535, passing central wavelength of 535 nm and width of 40 nm, Chroma, Bellows Falls, VT) is placed between the sample and the photodiode. A shift phase differential amplifier is designed to extract pulse signals of the fluorescence from the photodiodes. Another amplifier circuit is developed for PRS detection. An A/D converter (AD7707, Analog Devices, Inc., Norwood, MA, USA) and an ARM board (OK6410, Forlinx Embedded Technology. Inc., Hebei, China) form a data acquisition hardware. A QT program in Linux OS embedded in ARM board is used to acquire and process the signals from the amplifiers. The operation process of the developed system is shown in the supplementary information.

### Microfluidic Chip design and microfabrication

**Figure 2:**
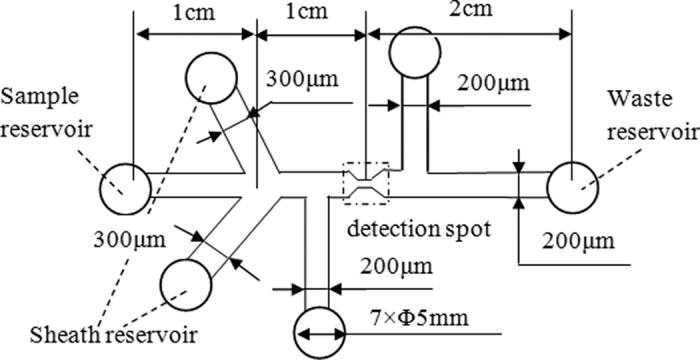
Diagram of structure and dimensions of the microfluidic chip used in this study.

The structural diagram of the designed microfluidic chip is shown in Fig. 2. This microfluidic chip has six reservoirs including one sample reservoir, one waste reservoir, two sheath reservoirs and two RPS reservoirs. The cell samples are put into the sample reservoir and the PBS buffer is placed into two sheath reservoirs for hydrodynamic flow focusing. The two laminar flow streams from the two sides will force the lymphocyte cells sample to move in a single line in the middle, and pass through the detection spot one by one along the main microchannel center line. The detection spot is the RPS sensing gate where the signals of fluorescence and RPS of the lymphocyte cells are detected simultaneously. The width and length of the main microchannel from the sample reservoir to the waste reservoir is 200 μm wide and 4 cm long, respectively. The width of the sensing gate is 15 μm. All the microchannels are 30 μm in height and all reservoirs have a diameter of 5 mm and a depth of 2 mm.

The microfluidic chip was fabricated by bonding a PDMS plate with a glass slide (24 mm × 50mm × 0.15 mm, Citotest Labware Manufacturing Co., Ltd., Haimen, China) by the following standard soft-lithography protocol^33^. A layer of SU-8 photoresist (MicroChem Co., Newton, MA) was spread on a bare silicon wafer (Lijing Co. Ltd., Quzhou, China) by a spin coater (G3P-8, Cookson Electronics Equipment, Indianapolis, IN). Then a photomask containing the designed microchannel pattern was mounted on the silicon wafer and excited with an OAI 150 illuminator. The SU8 master was attained after post-baking and developing processes. Liquid PDMS and curing agent was mixed, degassed and poured on the master, and then heated at 75 °C for 5 hours in a vacuum oven (Isotempmodel 280A, Fisher Scientific, Pittsburgh, PA) under normal pressure. Finally, the PDMS duplicate was taken from the master. Wells were formed by punching holes on the PDMS layer. The PDMS layer with the microchannel pattern was bound onto a glass slide after being treated for 60 seconds in a plasma cleaner (PDC-30G, Harrick Plasma, Ithaca, NY).

### Sample preparation

#### Lymphocyte cells preparation

1 mL fresh anti-coagulation human blood from healthy donors was mixed with 1 mL PBS buffer and 2 mL lymphocyte cells separation solution. The milky white lymphocyte cells at the second layer were obtained after the mixture was centrifuged for 15 minutes at 1800 revolutions/min. Then the lymphocyte cells were mixed with 5 mL cells washing liquid and centrifuged again for 20 min at 1800 revolutions/min. The 1 mL grey white cells precipitation was extracted and mixed with PBS buffer to obtain 5 mL lymphocyte cells samples.

#### UV Irradiation

The 5 mL lymphocyte cell sample was divided into 5 equal portions for blank control and irradiation under the four different radiation doses. A UVC light source (F6T5, 240 nm, Hitachi, Ltd. Japan) was used to irradiate the 1 mL cell samples in a culture dish. The radiation doses are 0 J/m^2^, 4 J/m^2^, 8 J/m^2^, 16 J/m^2^, and 32 J/m^2^, respectively.

#### Immunofluorescent labeling and assay

The immunofluorescent labeling of γ-H2AX with FITC was conducted by using the H2AX phosphorylation assay kit (Abcam, Cambridge, MA, USA) according to the manufacturer’s instructions. A commercial confocal microscopy (TCS SP5 II, Leica Microsystems GmbH, Wetzlar, Germany) was used for standard immunofluorescence assay. The analysis using the confocal microscopy was performed according to the standard operation manual.

## Results and Discussion

### Signal detection of fluorescent particles

**Figure 3:**
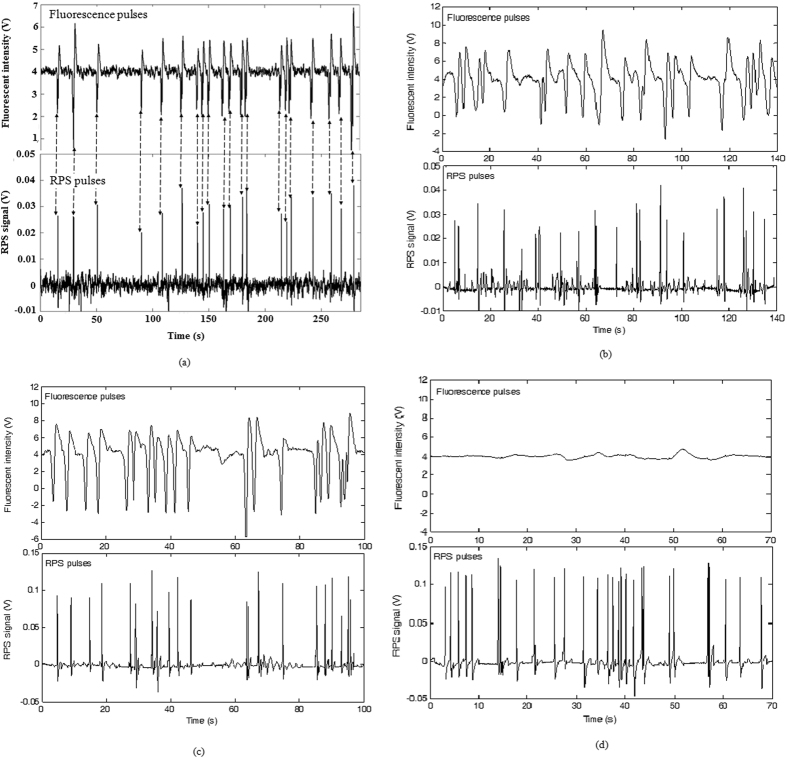
RPS and fluorescence signals of individual particles (**a**) (8.3 μm, fluorescent particle, 0.18% intensity, Dragon Green); (**b**) (5.8 μm, fluorescent particle, FICP-50-2); (**c**) (8.3 μm, fluorescent particle, 0.85% intensity, Dragon Green); (**d**) (10 μm, non-fluorescent particle, PPX-100-10).

In order to demonstrate that the developed system in this study can detect both the fluorescent signal and the RPS signal of the same particle simultaneously, four commercial polystyrene particles were chosen as sample to be tested. Typical signals of individual fluorescent particles (8.3 μm, fluorescent particle, 0.18% intensity, Dragon Green, Bangs Laboratories, IN, USA) are shown in Fig. 3(a). As can be seen from Fig. 3(a), a fluorescence pulse of a particle is generated when a fluorescent particle is passing through the detection spot. Correspondingly, a RPS pulse occurs. Every pulse represents a particle and the numbers of fluorescence pulses are equal to those of RPS pulses in Fig. 3(a). The amplitudes of both the fluorescence pulses and RPS pulses of the fluorescent particles fluctuate slightly, most likely due to the fluctuation in particle size. To clearly show the independence of both sensors of RPS and fluorescence detection, other three commercial polystyrene particles with different sizes and different fluorescent emissions were also tested using the developed microfluidic cytometer, the signals of individual particles are shown in Fig. 3(b) (5.8 μm, fluorescent particle, FICP-50-2, Spherotech, IL, USA), (c) (8.3 μm, fluorescent particle, 0.85% intensity, Dragon Green, Bangs Laboratories, IN, USA) and (d)(10 μm, non-fluorescent particle, PPX-100-10, Spherotech, IL, USA). As can be seen from these results, both sensors are independent and the average amplititude of PRS signals of the particles is proportional to the average size of the particles. While the average fluorescence intensity of the particles is related to the total intensity of the fluorescent dye coated on the surface of the particles. These results show that the developed microfluidic cytometer can reliably and independently detect the fluorescent and RPS signals of the same particles simultaneously.

### Signal analysis of lymphocyte cells after being radiated

**Figure 4:**
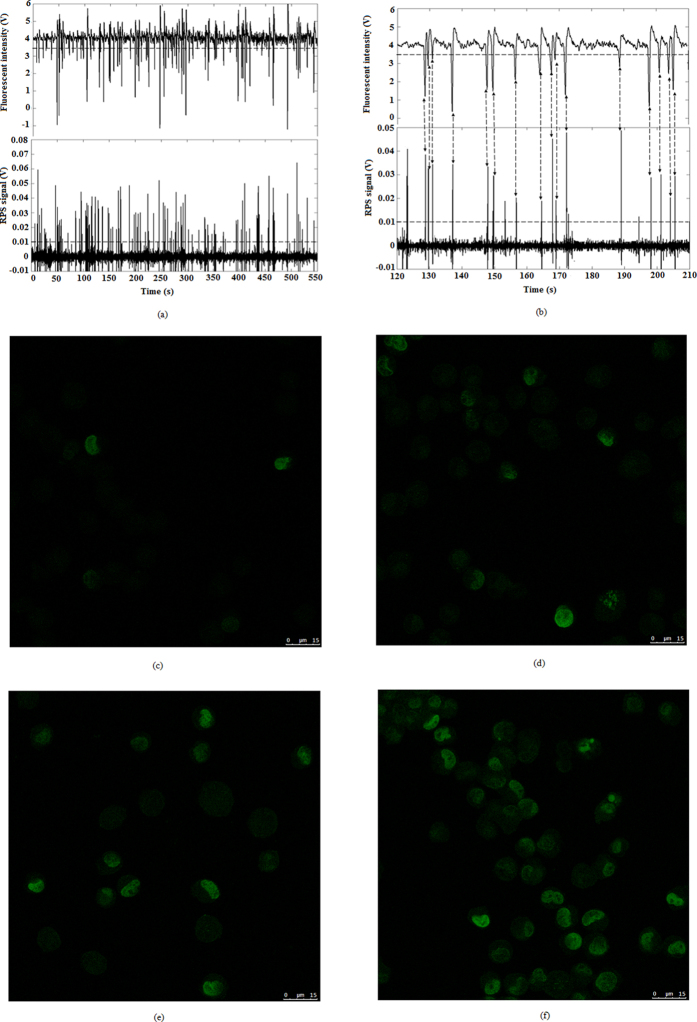
Typical signals of individual lymphocyte cells (**a**) radiated under 32 J/m^2^; (**b**) enlarged view of Fig. 4(a) from 120 s to 210 s. The actual confocal images of the γ-H2AX fluorescent marker in imaged cell radiated for different time under 32 J/m^2^ (**c**) for 1.25 minutes; (**d**) for 2.5 minutes; (**e**) for 5 minutes; (**f**) for 10 minutes.

To verify the performance of the developed microfluidic cytometer for evaluating the radiation damage, lymphocyte cells were adopted as sample to be assayed. Figure 4(a) shows the signals of the fluorescence and RPS of the lymphocyte cells after being radiated under 32 J/m^2^ and then stained by FITC fluorescent dyes. The results show that the amplitude of these pulses are not even because the degree of damage caused by the radiation for different cells is different so that the fluorescent intensities of lymphocyte cells are different. These different fluorescence intensities can be used to represent the damage distribution among the damaged cells. Theoretically, the fluorescence intensity of non-radiated cells is zero, however, it is worth noting that the real fluorescent intensity of non-radiated cells is not zero but a certain value owing to the diffusion of the fluorescent dye. Figure 4(b) shows that the corresponding voltage of the fluorescence intensity of non-radiated cells is less than 0.4 V, therefore, it is necessary to obtain a net fluorescence intensity by subtracting this value from the gross fluorescence intensity of the radiated cells as background noise. In order to show the peaks clearly, Fig. 4(b) shows an enlarged view of Fig. 4(a) from 120 s to 210 s. As seen in Fig. 4(b), each peak of fluorescence signals corresponds to a peak of RPS signals; however, not every RPS peak has a corresponding fluorescence peak. That is, the numbers of RPS signals–the total number of cells (N_RPS_) are more than those of fluorescence signals–the number of cells with radiation damage (N_fluo_). In other words, the ratio of these two numbers (N_fluo_/N_RPS_) can be used to measure the percentage of cells with radiation damage. To verify the differences in fluorescent intensity correlate with radiation damage, the actual confocal images of the γ-H2AX fluorescent marker in imaged cell under four typtical radiation conditions are given as comparison, which are shown in Fig. 4(c–f). From these actual images, we can see that the number of radiation damaged cells and the fluorescence intensity in cell both increase with the increase of the radiation intensity.

### Comparison experiments between the commercial confocal microscope and the developed microfluidic cytometer

**Figure 5:**
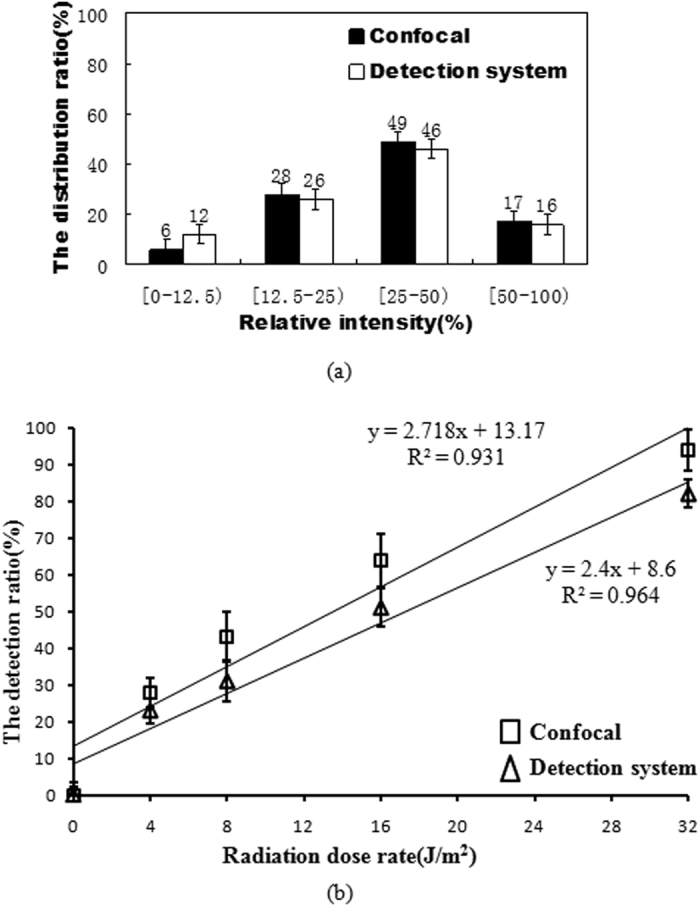
Comparison experiments between a commercial confocal microscope and the developed microfluidic cytometer. (**a**) Fluorescence intensity distribution for 100 cells after being radiated under 32 J/m^2^; (**b**) the ratio of the number of radiation damaged cells to the total number of cells in the sample at different radiation doses.

The fluorescence intensities of a group of lymphocyte cells after being radiated under 32 J/m^2^ were measured by a confocal microscope. When taking 100 cells that were detected to have fluorescent signals as sample, Fig. 5(a) shows their fluorescence intensities distribution. Under the same radiation treatment, the fluorescence intensities distribution of 100 cells that were detected to have fluorescent signals measured by the developed microfluidic cytometer is also shown in Fig. 5(a). The y-axis “the distribution ratio %” means the percent of the number of cells within a certain range of fluorescence intensity to the total number of cells. The results show that the fluorescence distributions measured by these two different instruments are consistent. Furthermore, it is easy to understand that the higher fluorescent intensity indicates stronger radiation damage. The developed microfluidic cytometer can detect the different degrees of damage among the radiation damaged cells. In Fig. 5(b), the y-axis “the detection ratio %” shows that the ratio of the number of radiation damaged cells to the total number of cells in the sample at the different radiation dosage rates. The results show that, with the increase of radiation dosage rate, the percentage of radiation damaged cells will increase. There is a good agreement between the results measured by the commercial confocal microscope and that by developed microfluidic cytometer.

## Conclusion

A handheld microfluidic flow cytometer has been developed in this work. The developed device is capable of evaluating the radiation damage of cells by measuring both the ratio of the number of cells with γ-H2AX fluorescence signals to the total numbers of cells in the sample and the distribution of γ-H2AX fluorescence intensities in the damaged cells. The number ratio indicates the percentage of the cells that are damaged by the radiation. The distribution in the fluorescent intensities points to different degrees of damage among the radiation damaged cells. The simple prototype of the developed microfluidic flow cytometer can detect fluorescent signals corresponding to a radiation dose rate as low as 0.95 J/m^2^, close to the detection limit of using a commercial confocal microscope, 0.75 J/m^2^. The developed device has many advantages such as low cost, easy operation and portability, and hence holds potential for onsite evaluation of radiation damage.

## Additional Information

**How to cite this article**: Wang, J. *et al.* A new hand-held microfluidic cytometer for evaluating irradiation damage by analysis of the damaged cells distribution. *Sci. Rep.* **6**, 23165; doi: 10.1038/srep23165 (2016).

## Supplementary Material

Supplementary Information
