# Supplementary material for: A new hand-held microfluidic cytometer for evaluating irradiation damage by analysis of the damaged cells distribution
Source: Sci Rep. 2016 Mar 17;6:23165. doi: 10.1038/srep23165 (PMC4794725; doi:10.1038/srep23165)
Supplement: Supplementary Information [file srep23165-s1.doc]

**Title:** A new hand-held microfluidic cytometer for evaluating irradiation damage by analysis of the damaged cells distribution

by Junsheng Wang, Zhiqiang Fan, Yile Zhao, Younan Song, Hui Chu, Wendong Song, Yongxin Song, Xinxiang Pan, Yeqing Sun and Dongqing Li

Screen shots from operation video in order

1.
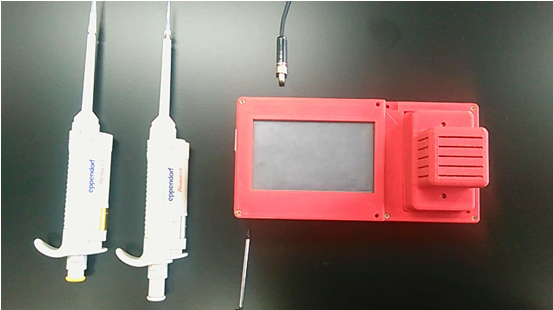

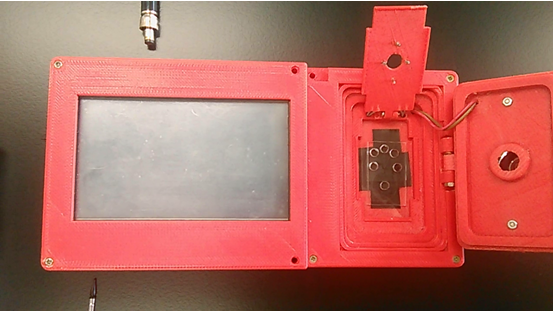
Put the device on the desk
2. Put a microfludic chip in the device(in dashed frame)
3.
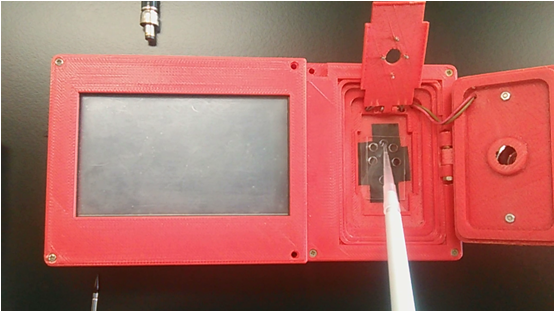
Add sample and buffer solution in the microfludic chip(in dashed frame)
4.
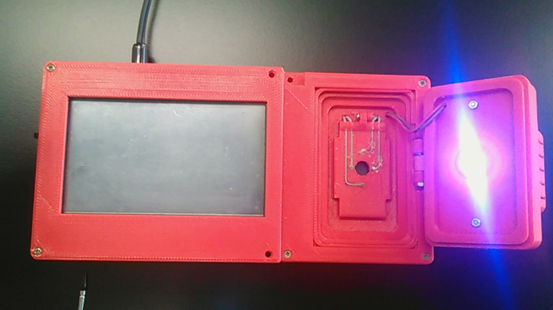
LED light source on(in dashed frame)
5.
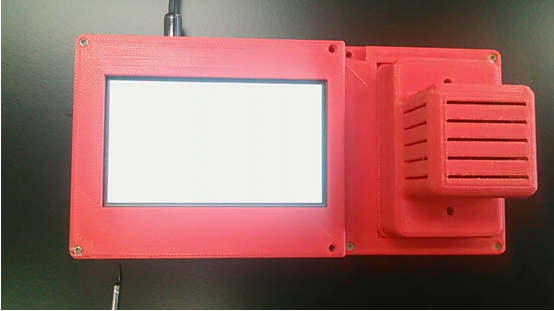
Power on and system starts( switch in dashed frame)
6.
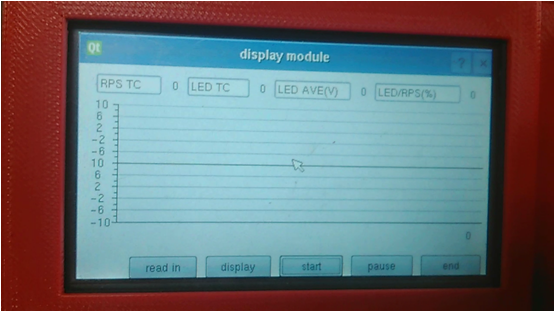
The interface on display screen(in dashed frame)
7.
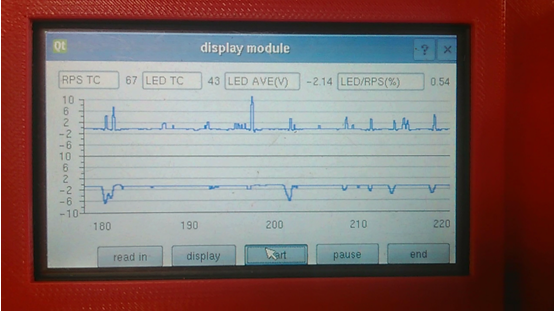
The measurement starts and the software in ARM
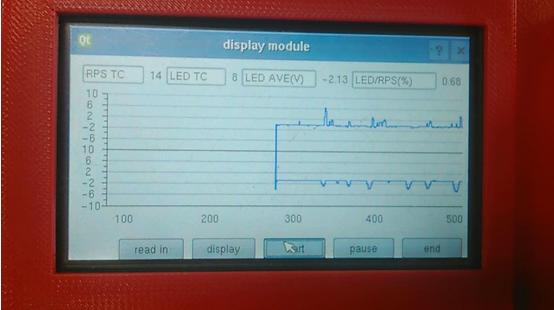
works(in dashed frame)
8. The detection results are showing on the screen(in dashed frame)
